# Supplementary material for: MetaRibo-Seq measures translation in microbiomes
Source: Nat Commun. 2020 Jun 29;11:3268. doi: 10.1038/s41467-020-17081-z (PMC7324362; doi:10.1038/s41467-020-17081-z)
Supplement: Supplementary file 10 — Supplementary Data 7 [file 41467_2020_17081_MOESM10_ESM.zip › File2/Confidence_VeryHigh_Taxonomy/156389_out.krona.html]

Javascript must be enabled to view this page.

members
magnitude
magnitudeUnassigned
count
unassigned
taxon
rank

156389\_out

8

superkingdom
2
8

8
201174
phylum

8
class
1760

8
85004
order

31953
family
8

genus
1678
8

1
216816
species

subspecies

SRS143466\_contig\_number\_30044
1682
1

2
species

SRS012902\_contig\_number\_contig-100\_7275.7275SRS105082\_contig\_number\_14417
28026


SRS013638\_contig\_number\_12067SRS018656\_contig\_number\_contig-100\_1676.1676SRS050752\_contig\_number\_28029SRS1055056\_contig\_number\_12981SRS893341\_contig\_number\_contig-100\_5118.42251
1680
species
5
